# A Complex Study of Nuclear Magnetic Resonance for Olefin Polymerization Catalyst

**DOI:** 10.3390/polym18111304

**Published:** 2026-05-26

**Authors:** Xiaojie Ji, Xuelei Duan, Xinyue Liu, Yulian Li, Shan Ye, Fuyue Tian, Yu Zhou, Congyun Liu, Linge Ma, Shiyi Wu, Wenhua Sun, Zhe Zhou

**Affiliations:** 1 National Institute of Clean-and-Low-Carbon Energy, Beijing 102209, China; 2 Tsinghua University, Beijing 100084, China; 3 Institute of Chemistry, Chinese Academy of Sciences, Beijing 100190, China

**Keywords:** polyolefin, olefin polymerization, catalyst, in situ NMR

## Abstract

This review summarizes recent applications of nuclear magnetic resonance (NMR) in olefin polymerization catalysis. Due to its capability for quantitative characterization of molecular structures and in situ study, NMR is employed to study the structure of catalysts, and to trace catalyst/cocatalyst interactions, the evolution of active species, monomer insertion, and chain-end formation. This review emphasizes the activation mechanisms of molecular catalysts, ion-pair structures, and the measurement of kinetics. It also discusses the potential applications of in situ multinuclear NMR and isotope labeling technologies in olefin polymerization catalysis studies.

## 1. Introduction

Polyolefins are important materials in modern industry and daily life due to their wide application, durability and low cost. They are the most consumed synthetic polymer materials globally, with production exceeding 230 million tons in 2024 [1,2,3,4,5]. Except for low-density polyethylene produced via free radical polymerization of ethylene under extreme pressure and high temperature using peroxide initiators, most polyolefins are synthesized using catalysts. Olefin polymerization catalysts can be broadly categorized into oxide catalysts (e.g., Phillips catalysts) [6,7,8], Ziegler–Natta catalysts [9,10,11] and molecular catalysts [12]. According to the type of active metal, they can also be classified into early-transition metal catalysts [13,14] and late-transition metal catalysts [15,16,17,18,19]. Oxide catalysts mainly refer to Phillips catalysts, whose primary chemical composition consists of CrOx-based compounds supported on SiO_2_. Ziegler–Natta catalysts are mainly composed of TiClₓ supported on MgCl_2_, SiO_2_, or their mixed carriers. Both are typical supported catalysts, featuring diverse active-site structures and the characteristics of multi-site catalysts. Molecular catalysts are composed of a central metal and ligands, usually with well-defined molecular structures and uniform active sites, thus exhibiting single-site properties. However, molecular catalysts can also show multi-site catalytic behavior after immobilization [20,21,22]. In practical catalytic systems, complex interactions occur among various chemical species. In addition to the aforementioned supports and active metal species, cocatalysts, electron donors, counterions, and other components are usually required [23]. A thorough understanding of the catalytic mechanism during reactions relies on in-depth characterization of the molecular structures and interactions of these key components.

Nuclear magnetic resonance (NMR) has long been used to characterize polyolefin molecular structures [24,25] and has gradually become a standardized method for studying polyolefin molecular sequence structures [26,27,28,29]. Direct observation of catalyst systems using NMR is an intrinsically attractive analytical strategy [30,31,32,33]. It can provide molecular-level structural information of catalytic systems, characterize direct interactions between precatalysts and cocatalysts, and reveal structural evolution during activation [34,35,36]. Furthermore, NMR enables in situ monitoring of polymerization mechanisms, capturing real-time spectral changes to uncover key processes including reaction kinetics, active-site formation, and monomer insertion pathways [37,38,39,40,41]. Nevertheless, significant challenges have long persisted due to the high structural complexity of polymerization catalysts, paramagnetic characteristics of some samples, and quadrupolar moments of relevant nuclei. With recent advances in NMR technology, these obstacles have been gradually overcome, unlocking substantial research potential. Focusing on recent progress in this field, this review summarizes State-of-the-Art applications of NMR in characterizing the molecular structure, activation mechanism, ion-pair structure, and polymerization kinetics of polymerization catalysts. Based on these advances, future prospects are also discussed.

## 2. Supported Catalysts

### 2.1. Phillips Catalyst

#### 2.1.1. Current Research Progress

The Phillips catalyst is an important industrial ethylene polymerization catalyst with chromium oxide (CrOx) as the active component supported on amorphous silica gel [42,43]. Discovered by the Phillips Petroleum Company in the early 1950s, it is still the predominant catalyst system for producing high-density polyethylene (HDPE), accounting for 40–50% of the global HDPE market, with an annual production of tens of millions of tons [44,45,46,47,48]. The uniqueness of the Phillips catalyst originates from the complex surface chemistry of amorphous silica supports [45]. There are various types of silanol groups on the silica surface, including isolated silanol (≡SiOH), vicinal silanol (Si–OH groups on two adjacent silicon atoms linked by hydrogen bonds), and geminal silanol (≡Si(OH)_2_). Chromium species bind to surface silanol groups through esterification to form surface species such as monochromate, dichromate or polychromate [49]. After high-temperature calcination (usually 500–900 °C), chromium exists in the form of hexavalent chromium (Cr(VI)) and is reduced to active centers (generally believed to be Cr(II)) in an ethylene atmosphere to initiate polymerization [50].

The most prominent feature of the Phillips catalyst is that it can be directly activated by ethylene without cocatalysts, which is different from Ziegler–Natta catalysts and metallocene catalysts that require alkyl aluminum cocatalysts [51]. The polyethylene produced has unique microstructure characteristics: ultra-wide molecular weight distribution (MWD > 10), which enhances processability; a long-chain branched structure that imparts excellent mechanical properties to the final products; and unsaturated chain ends, enabling functionalization modifications. To optimize catalyst performance, various industrial modification strategies have been developed, including: titanium modification, where the introduction of TiO_2_ significantly increases polymerization activity, broadens the molecular weight distribution toward the low-molecular-weight region, modulates comonomer distribution, and enhances the regioselectivity of 2,1-insertion [46]; alkylaluminum modification, where the addition of alkylaluminum cocatalysts such as triethylaluminum (TEA) regulates the formation of active centers and optimizes polymerization kinetic behavior [47]; and fluorination modification, which influences the acidity of the support and thereby affects the electron density of the active centers. Solid-state NMR, especially ^1^H MAS NMR and ^29^Si CP/MAS NMR, has become an important tool to reveal the surface chemistry of Phillips catalysts due to its high sensitivity to surface proton species and silicon framework structures [44,45].

Cheng et al. [45] systematically studied the types, contents and chemical environment changes in surface proton species on silica gel (SG) and Phillips catalysts (PC) under different calcination temperatures (120–800 °C) using ^1^H MAS NMR. The ^1^H MAS NMR spectra (Figure 4 on page 5 in Cheng et al.’s work [45]) show that all samples have a main peak at 1.7–1.8 ppm, attributed to the protons of surface silanol (Si–OH). Two rotational sideband satellite peaks appear on both sides of the main peak at 12 ppm and −10 ppm. Studies have demonstrated that the main peak actually consists of two overlapping signals: the broad peak is attributed to hydrogen-bonded silanol groups, while the narrow peak corresponds to isolated silanol groups [45,52]. With the increase in calcination temperature, the broad peak of hydrogen-bonded silanol gradually weakens, and the main peak gradually narrows, indicating that the hydrogen bond interaction between surface hydroxyl groups weakens and the proportion of isolated hydroxyl groups increases. After calcination at 600 °C (PC600 and SG600), hydrogen-bonded hydroxyl groups are almost completely removed, and surface silanol groups are basically converted to an isolated state.

By comparing the ^1^H chemical shifts in catalysts and pure silica samples, it is found that the silanol chemical shifts (about 1.80 ppm) of catalyst samples (PC200, PC300, PC400, PC600) are about 0.06 ppm higher than those of pure silica samples (about 1.74 ppm) after calcination at 200–600 °C [45]. This small but systematic shift difference indicates that surface chromium species have an electron-withdrawing inductive effect on adjacent silanol groups, changing the electron cloud density of hydroxyl protons. This result is consistent with the findings of Nishimura and Thomas [45,52] based on CrO_2_Cl_2_ model catalysts, who also observed spectroscopic evidence for the interaction between silanol groups and low-valent chromium ions.

Figure 5 on page 5 in Cheng et al.’s work [45] illustrates the variation in the chemical shift in silanol groups as a function of calcination temperature. For pure silica calcined above 200 °C, the chemical shift remains stable at approximately 1.75 ppm, whereas that of the catalyst samples is maintained near 1.80 ppm [45]. This persistent difference further confirms that surface chromate species hinder the removal of partial silanol groups during high-temperature calcination, thereby enabling a small fraction of silanols to remain even after thermal treatment at 800 °C.

For uncalcined raw silica (raw SiO_2_), the ^1^H spectrum exhibits a broad main peak at 3.90 ppm, which is assigned to strongly hydrogen-bonded physisorbed water confined within micropores [45]. Similarly, the broad peak of the catalyst precursor PC120 at 3.29 ppm also originates from residual physisorbed water, which may partially persist within the microporous structure even at elevated temperatures. Accordingly, ^1^H MAS NMR results not only reveal the regulatory effect of calcination temperature on the type and content of surface hydroxyl groups but also, more importantly, provide direct evidence for the electronic interaction between chromium species and silanol groups, as well as the steric hindrance effect of chromate species on the high-temperature dehydroxylation process. These findings offer critical experimental insights into the anchoring mechanism of chromium species during the preparation of Phillips catalysts.

A 2026 study systematically compared chromium catalysts supported on two commercial silica carriers, namely Grace-Davison-955 and AGC-DM-302 [42]. This research highlights the condition dependence of polymerization behavior, wherein polymer chain growth is co-regulated by reaction kinetics and mass transfer processes. This conclusion complements the electronic effect of chromium species on silanol groups reported by Cheng et al. [45]. Specifically, Cheng’s work focused on surface chemistry during catalyst preparation, while this study emphasized mass transfer and chain propagation mechanisms in the catalytic reaction stage.

In 2024, Kim et al. [53] adopted an importance sampling learning algorithm to investigate the site-averaged kinetics of Phillips catalysts on an amorphous silica slab model for the first time. Notably, this study disproved the applicability of the Bell–Evans–Polanyi principle in explaining the activity discrepancy among active sites of Phillips catalysts, and demonstrated that the divergent effects of strain energy on intermediates and transition states dominate the origin of site-dependent catalytic activity.

Parab et al. [43] comprehensively investigated the influence of trace oxygen (50–500 ppm) on the induction period of Cr(VI)/SiO_2_ catalysts. Their findings are of great significance for the regulation of industrial polymerization processes. As a ubiquitous impurity, fluctuating oxygen concentrations substantially alter catalyst activation behavior and final polymer quality.

#### 2.1.2. Summary and Outlook

At present, the research directions of Phillips catalyst mainly focus on precise identification of active sites, support effects and structure–activity relationships, initiation and chain growth mechanisms, industrial process optimization and development of new catalytic systems. The research is undergoing a paradigm shift from “static structure characterization” to “dynamic process analysis” and from “empirical optimization” to “theoretically guided design”. The in-depth integration of modern spectroscopic methods (operando XAS, EPR, NMR) and computational simulations (DFT, machine learning, reaction kinetic models) is gradually uncovering the mechanistic mystery of this industrial catalyst over sixty years. However, the final confirmation of active center valence, the universal law of support effects, and rational catalyst design for high-performance polyolefins remain core challenges for future research.

### 2.2. Ziegler–Natta Catalysts

#### 2.2.1. Current Research Progress

Ziegler–Natta catalysts (ZNCs) are one of the most important catalyst systems in the polyolefin industry, widely used in industrial production of polyethylene and polypropylene [54]. Since first discovered by Karl Ziegler and Giulio Natta in the 1950s, ZNCs have experienced decades of development and evolution and become the cornerstone of modern catalytic polymerization technology [55,56]. Standard ZNCs consist of four core components. The primary catalyst is usually a transition metal compound such as titanium tetrachloride (TiCl_4_), which serves as the precursor for catalytic active sites [57,58]. Magnesium chloride (MgCl_2_) is predominantly adopted as the support; its crystal structure is similar to that of titanium trichloride (TiCl_3_), enabling efficient dispersion and stabilization of titanium active centers [59,60]. Cocatalysts represented by trialkylaluminum (AlR_3_) reduce tetravalent titanium and facilitate the formation of Ti-C active bonds [61,62]. Electron donors are divided into internal and external types, which are critical for regulating the stereoselectivity of catalysts. Common internal electron donors include esters (e.g., diisobutyl phthalate, DiBP), ethers (e.g., 2,2-dimethyl-1,3-dimethoxypropane, DMDOMe), and alcohols (e.g., ethanol and tetrahydrofuran) [61,63]. External electron donors, typically alkoxysilanes, are added together with cocatalysts to selectively poison atactic active sites and thereby enhance the isotacticity of resultant polymers.

Although ZNCs have been industrially applied for decades, the structure and formation mechanism of their active centers remain an area of intense research requiring continuous in-depth exploration. This fundamental challenge primarily stems from multiple factors. First, the inherent complexity of the catalytic system. ZNCs are multicomponent and heterogeneous systems with an extremely low content of active centers, where the titanium loading is generally only 2–4 wt%. Second, the structural disorder of the support. After mechanical ball milling or chemical activation, MgCl_2_ exhibits a highly disordered microstructure with abundant crystal defects [64]. Third, the diversity of surface active sites. Titanium species may exist in the form of mononuclear, dinuclear or cluster structures on different crystal facets and defect sites of MgCl_2_. Finally, the direct characterization of active centers faces great difficulties. Most catalytically active Ti(III) centers are paramagnetic, making them undetectable by conventional characterization techniques [65].

Owing to the aforementioned complexity of ZNCs, conventional characterization methods encounter substantial limitations in elucidating their microscopic structures. Solid-state NMR has become a powerful tool to study this complex system due to its sensitivity to local chemical environment, no requirement for long-range order, and recent advances in high magnetic field, fast high-resolution magic angle spinning (HR-MAS), and multi-quantum pulse sequences. Through direct observation of quadrupolar nuclei such as ^47/49^Ti, ^25^Mg, ^35^Cl, and ^27^Al combined with DFT calculations, solid-state NMR can provide atomic-level information on catalyst surface sites, electron distribution, coordination symmetry, and donor–support interactions.

Iijima et al. systematically studied the adsorption behavior and local structure of TiCl_4_ on different crystal planes of MgCl_2_ support using high-field (21.8 T) ^47/49^Ti solid-state NMR combined with periodic DFT calculations [58].

**Figure 1:**
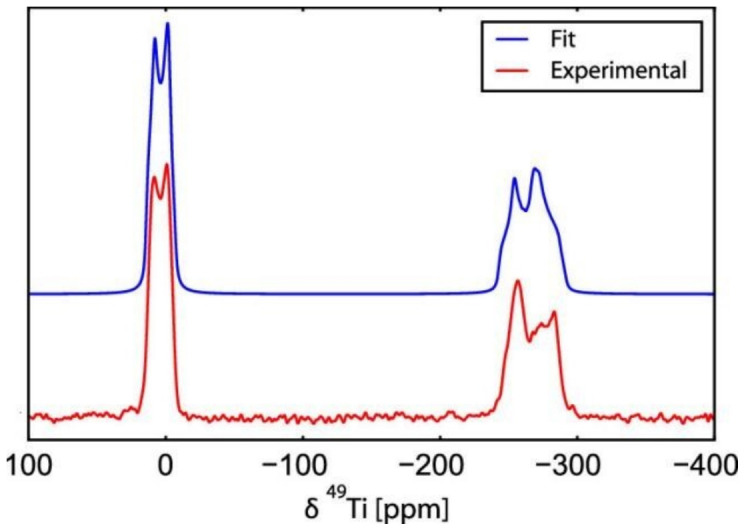
Static ^47/49^Ti NMR spectrum of frozen TiCl_4_ (red) obtained by single-pulse excitation. The results from a simultaneous fit of the ^47^Ti and ^49^Ti resonance, taking into account quadrupolar interaction and CSA, are shown in blue. Reproduced from [57].

Studies have shown that the ^47/49^Ti MAS NMR spectra of unground samples are nearly identical to those of liquid, indicating that titanium species remain in a free state. In contrast, after 20 h of mechanical grinding, the NMR spectra of the samples (Figure 1 on page 3 in Iijima et al.’s work [58]) exhibit significant broadening and high-field shifting, displaying the typical characteristics of disordered materials. This demonstrates that strong interactions occur between the core and the surface, leading to the formation of adsorbed species with locally disordered structures.

The experimental spectra were fitted using the Czjzek distribution model, yielding key NMR parameters including an average quadrupole product P_Q_ = 6.55 + 0.89 MHz and isotropic chemical shift (δiso = −2.8 ± 7.6 ppm). DFT calculations were further performed to construct adsorption models of TiCl_4_ on the MgCl_2_ (110), (104), and (104)-step defect surfaces, with the corresponding ^47/49^Ti NMR parameters systematically calculated. The results reveal that the theoretically predicted NMR parameters are in excellent agreement with experimental values (Figure 6b on page 6 in Iijima et al.’s work [58]) only when monomeric TiCl_4_ is weakly adsorbed on the (104) surface and maintains an approximately tetrahedral geometry. In contrast, Ti species anchored on the (110)- and (104)-step defect surfaces adopt distorted octahedral TiCl_6_ configurations, whose quadrupole coupling constant C_Q_ and chemical shift (δiso) deviate markedly from experimental data (Figure 6a,c on page 6 in Iijima et al.’s work [58]). This work demonstrates that solid-state NMR enables direct characterization of the local coordination environment of surface Ti sites in ZNCs. Combined with DFT simulations, it can reliably identify that TiCl_4_ is preferentially adsorbed on the (104) facet of MgCl_2_. This fully highlights the unique advantages and great potential of solid-state NMR in elucidating the surface structure of active-site precursors in ZNCs.

Blaakmeer et al. employed high-field (20.0 T) solid-state NMR spectroscopy, combined with ^47/49^Ti and ^35/37^Cl multi-nuclear detection, the QCPMG signal enhancement pulse sequence, and periodic DFT calculations, to systematically investigate the local structure and coordination environment of titanium active-site precursors in Ziegler–Natta catalysts [57].

The authors first established benchmark parameters using titanium chloride model compounds. The solid-state ^47/49^Ti NMR spectra of solid TiCl_4_ (Figure 1) exhibit characteristic quadrupolar line shapes for both isotopic resonances. Fitting results yield a ^49^Ti quadrupole coupling constant C_Q_ = 1.13 MHz. This confirms that Ti centers with nearly tetrahedral symmetry can still generate detectable quadrupolar interactions, and demonstrates that Ti NMR is highly sensitive to local structural symmetry.

**Figure 2:**
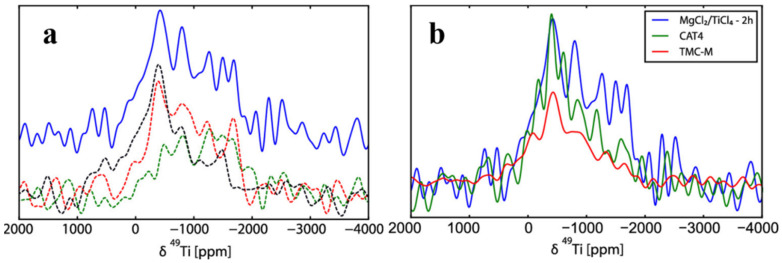
(**a**) Static ^47,49^Ti QCPMG spectrum of the 2 h ball-milled MgCl_2/_TiCl_4_ adduct (blue), sum of three spectra acquired at offsets of 0, −800, and −1400 ppm (dashed lines); (**b**) Static ^47,49^Ti QCPMG spectra of the 2 h ball-milled adduct (blue), CAT4 (green), and TMC-M (red). Reproduced from [57].

On this basis, the research was further extended to practical catalyst systems. The ^47/49^Ti spectrum of the MgCl_2_/TiCl_4_ adduct after 2 h of ball milling (Figure 2a) exhibits an extremely broad linewidth of 110 kHz, which is much larger than that of the wet-impregnated sample. Continuous acquisition with multiple frequency offsets is required to record the full spectral range, demonstrating that ball milling induces strong interactions between Ti species and the support and thereby generates a highly asymmetric coordination environment. This observation is consistent with the surface adsorption models predicted by DFT, such as the six-coordinate Corradini sites.

Further comparison of the Ti spectra of the industrial precatalyst CAT4 containing the electron donor di-n-butyl phthalate (DnBP) and the TMA-activated ternary system TMC-M (Figure 2b) reveals that the spectral profile of CAT4 is nearly identical to that of the ball-milled adduct. This indicates that the DnBP electron donor is not directly coordinated around the Ti centers. In contrast, the TMC-M sample shows a remarkable reduction in signal intensity, suggesting that partial Ti species are reduced by TMA to form NMR-silent Ti^3+^ species. Nevertheless, the line shape of the residual Ti signals remains unchanged, implying that most Ti sites are not directly modified by the activator.

This study verifies that solid-state NMR can directly probe the local coordination environment and symmetry of Ti centers in Ziegler–Natta catalysts, and track the structural evolution of Ti species from model compounds to practical catalytic systems. It further reveals the strong surface interactions induced by ball milling, the non-coordinating behavior of electron donors, and the selective reduction effect of alkylaluminum. These findings provide atomic-level experimental evidence for clarifying the structural nature of pre-active sites in ZNCs and the structural origin of the low proportion of ultimate active centers.

The aforementioned studies are of great significance in establishing solid-state NMR characterization methods for Ti centers, distinguishing the NMR parameters of Ti^4+^ in diverse coordination environments, and assigning adsorption sites in combination with DFT calculations. However, restricted by the limited resolution of solid-state NMR for quadrupolar nuclei, static measurement conditions, the simplification of model systems, and the absence of direct observation of paramagnetic active centers, these works still fail to fully address the core scientific issue regarding the structure and formation mechanism of active sites in Ziegler–Natta catalysts.

In another investigation reported by Blaakmeer et al. [61], high-field solid-state NMR techniques, including ^1^H MAS, ^13^C CPMAS, ^1^H–^13^C HETCOR and ^1^H DQ-SQ two-dimensional correlation spectroscopy, were combined with periodic DFT calculations to systematically explore the coordination structure and adsorption sites of three types of industrial internal electron donors in ZNCs on the surface of the MgCl_2_ support. The investigated donors cover DMDOMe (1,3-diether, Do1), DMFluo (fluorenyl diether, Do2), and DiBP (phthalate, Do3).

**Figure 3:**
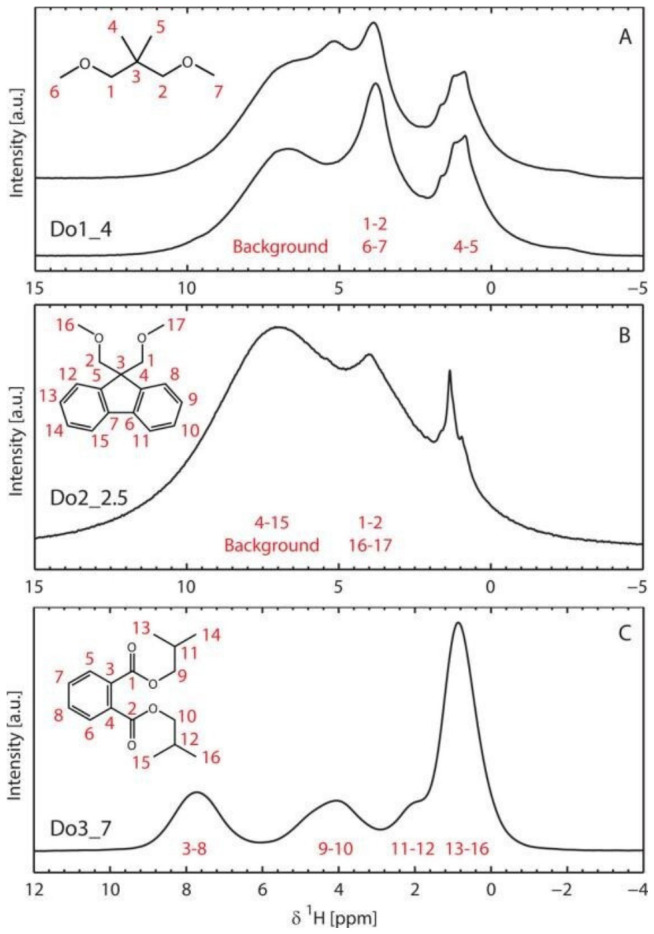
^1^H MAS NMR spectra of (**A**) DMDOMe in Do1_4, (**B**) DMFluo in Do2_2.5, and (**C**) DiBP in Do3_7. Spectra are acquired using (**A**) 15, (**B**) 20, and (**C**) 50 kHz MAS at B0 of (**A**) 9.4 and (**B**,**C**) 20.0 T. The bottom trace in (**A**) shows a fresh sample of Do1_4, while the top trace shows a hydrated sample. Reproduced from [61].

**Figure 4:**
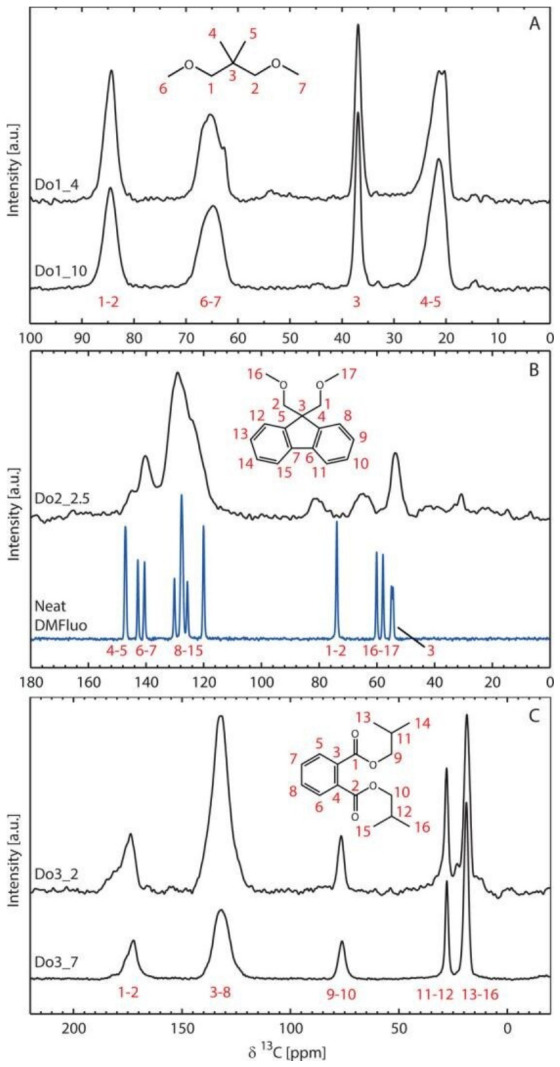
^13^C CPMAS NMR spectra of binary adducts obtained at 9.4 T, where DMDOMe adducts are shown in (**A**), neat DMFluo (blue) and Do2_2.5 (black) in (**B**), and DiBP adducts in (**C**). Reproduced from [61].

Experimental results reveal that the ^1^H MAS spectra (Figure 3) show no obvious water signal for all binary adduct samples. Distinct H_2_O peaks only appear after exposure to ambient air (Figure 3A), which confirms that electron donors can effectively cover the unsaturated sites on the support surface and inhibit water adsorption. The ^13^C CPMAS spectra (Figure 4) serve as the key evidence in this study. For the DMDOMe adduct (Figure 4A), the chemical shifts of oxygen-linked carbon groups (–OCH_2_– and –OCH_3_) exhibit a remarkable downfield shift (>5 ppm) compared with neat DMDOMe, and splitting is observed for methyl and methoxy signals. These phenomena indicate that DMDOMe coordinates with Mg sites via oxygen atoms and forms asymmetric surface structures. For the DMFluo adduct (Figure 4B), the signals of –OCH_2_– and –OCH_3_ undergo considerable shifts and multiplet splitting, while only minor variations are detected in the aromatic region. In the case of the DiBP adduct (Figure 4C), three well-resolved carbonyl carbon signals at 172.5, 175.8 and 182.0 ppm are identified. Their relative intensities vary with DiBP loading content, directly reflecting the adsorption distribution of DiBP on different crystal facets.

Figure 1 and Figure 2 focus on the existence form and activation mechanism of Ti centers on the support, while Figure 3 and Figure 4 investigate the binding mode of electron donors with the support and their stabilization effect on specific crystal facets. Collectively, these studies clarify the structural nature of pre-active sites in ZNCs. Nevertheless, neither work explores the synergistic interaction between Ti centers and electron donors, nor achieves simultaneous NMR observation of Ti species and electron donors in activated systems. This constitutes a critical research gap and represents an important direction for future development.

In the studies on Phillips and Ziegler–Natta catalysts, the applied solid-state NMR techniques—^1^H MAS/^29^Si CP/MAS for the former and ^47/49^Ti QCPMG for the latter—offer complementary but distinct insights, each with inherent limitations. The ^1^H and ^29^Si methods excel at characterizing surface silanol chemistry and their electronic interaction with chromium, yet they are inherently static, cannot detect the paramagnetic Cr(II) active sites, and provide only indirect evidence of the chromium coordination environment. Conversely, ^47/49^Ti NMR coupled with DFT modeling directly probes the local geometry of Ti^4+^ precatalyst sites on MgCl_2_, enabling identification of specific adsorption facets, but it remains blind to the actual Ti^3+^ active centers (NMR-silent due to paramagnetism) and relies on simplified static models that exclude cocatalysts, electron donors, and dynamic activation processes. Across both catalyst systems, a common critical shortcoming persists: no in situ observation under real polymerization conditions is achieved, and DFT-assisted interpretations are constrained by idealized surface representations, leaving the atomic-scale evolution of true active species largely inaccessible.

#### 2.2.2. Summary and Outlook

At present, most solid-state NMR studies focus on TiCl_4_/MgCl_2_ binary systems, lacking direct spectroscopic evidence for the synergistic effects of internal electron donors, cocatalysts and titanium centers in real catalysts. In situ and quantitative studies on the coverage, distribution and dynamic exchange behavior of electron donors on a MgCl_2_ surface are insufficient. Paramagnetic NMR studies on active-center Ti^3+^ are still in the initial stage, making it difficult to directly observe its evolution during polymerization.

Future development directions: (1) Develop multinuclear (^25^Mg, ^35^Cl, ^47,49^Ti, ^13^C) in situ solid-state NMR to monitor the dynamic changes in electron donors and titanium centers in real time under reaction conditions. (2) Combine ultra-high field (≥35 T) solid-state NMR to improve the resolution and sensitivity of quadrupolar nuclei (such as ^49^Ti). (3) Carry out ^13^C or ^2^H isotope-labeled electron donors to detect the spatial proximity and coordination mode between electron donors and MgCl_2_ surface using CPMAS or REDOR techniques. (4) Combine DFT calculations and molecular dynamics simulations to construct structural models closer to real catalyst surfaces.

### 2.3. Supported Molecular Catalysts

#### 2.3.1. Current Research Progress

Solid-state NMR technology, especially the combination of magic angle spinning (MAS) and cross-polarization (CPMAS), has become an important means to study the surface structure and active center formation mechanism of supported metallocene catalysts. Atiqullah et al. [66] systematically used ^29^Si CPMAS NMR and ^27^Al MAS NMR to characterize metallocene catalysts supported on silica gel PQ 3030, revealing the evolution of surface functional groups, the coordination environment of the cocatalyst methylaluminoxane (MAO), and their interactions with zirconium centers.

^29^Si CPMAS NMR is an effective tool for analyzing the surface chemical structure of silicon-based supports. Atiqullah et al. [66] found three different types of silicon species on the surface of untreated silica gel: geminal silanol groups ((HO)_2_–Si–(O–Si)_2_) with an isotropic chemical shift at −91.31 ppm, isolated silanol groups (HO–Si–(O–Si)_3_) resonating at −101.26 ppm, and a siloxy group (Si–(O–Si)_4_) showing a chemical shift of −110.47 ppm. After dehydroxylation at 250 °C, the signal intensity of geminal and isolated silanol decreased, while the siloxy group signal increased, indicating that some silanol groups underwent condensation reactions to form siloxane structures.

Further investigations reveal that after the reaction of dehydroxylated silica with MAO or ^n^BuGeCl_3_, geminal silanol groups are also involved in the surface reaction. This result indicates that silanol groups, especially geminal and isolated silanols, act as the dominant active sites for the chemical bonding of MAO and Ge compounds, laying a structural foundation for subsequent catalyst immobilization.

^27^Al MAS NMR was used to study the coordination state changes in MAO before and after loading. The results show that there are three obvious resonance peaks in the ^27^Al NMR spectrum of dried MAO, located at 3.22 ppm, 33.27 ppm, and 69.23 ppm, respectively, with hydrated Al^3+^ as the reference, indicating that aluminum atoms in MAO exist in various coordination environments, supporting the cage structure model of MAO. Specifically, MAO is constructed by the fusion of (MeAlO)_6_ units and is capable of encapsulating TMA molecules.

When Cp_2_ZrCl_2_ was immobilized onto MAO-modified silica, broadened resonance peaks appeared in the ^27^Al NMR spectrum, with chemical shifts shifting to −32.41 ppm, 3.09 ppm, and 52.08 ppm, respectively. These signals exhibited an obvious high-field shift compared with those of dried MAO. Such variations may result from multiple factors, including the methylation of Cp_2_ZrCl_2_ by MAO, the coordination of Zr^4+^ with MAO, and the resulting electronically deficient solid-state environment. These observations further confirm the formation of cationic zirconium active centers. The above findings provide direct experimental evidence for understanding the functional mechanism of MAO as a cocatalyst in supported catalytic systems.

#### 2.3.2. Latest Progress

In recent years, with the development of solid-state NMR, dynamic nuclear polarization (DNP) enhancement technology and ultra-high field magnets, significant progress has been made in the research of supported molecular catalysts.

(1) Breakthrough in direct NMR detection technology of transition metal nuclei

In 2025, Koppe et al. [67] first demonstrated the powerful ability of ^195^Pt solid-state NMR in characterizing Pt site coordination environments in single-atom catalysts (SACs). This study combines static and MAS ^195^Pt NMR techniques to acquire full-range NMR spectra of samples with an ultra-low Pt content of only 1 wt% within a reasonable acquisition time. More importantly, Monte Carlo simulations were adopted to convert the original NMR spectra into “SAC signals” that characterize the local coordination environment, enabling the quantitative evaluation of the distribution and homogeneity of Pt active sites. This approach can track the effects of synthetic parameters and monitor structural evolution during reactions, thereby offering critical insights for the rational and repeatable fabrication of SACs with targeted structures.

(2) Deepening of multinuclear solid-state NMR in metal-support interaction research

In 2025, Berkson et al. [68] systematically summarized the latest progress of solid-state NMR combined with DFT calculations in understanding the local environment of metal centers in homogeneous and heterogeneous catalysts. The study focused on how NMR parameters of transition metal nuclei such as ^95^Mo, ^195^Pt, ^109^Ag, ^183^W, ^51^V and ^47/49^Ti provide unique information on metal site electronic structure and coordination environment. Analysis of the origin of the chemical shift tensor through DFT calculations enables the correlation of NMR signals with specific local coordination environments, frontier molecular orbitals, and corresponding reactivity, thereby establishing structure–activity relationships in catalytic systems.

#### 2.3.3. Summary and Outlook

Existing studies mostly focus on static supported models, lacking in situ NMR monitoring of the dynamic bonding process between catalysts and supports during loading. There are few studies on the structural evolution and active center formation mechanism of supported catalysts during activation. A molecular-level understanding of the interaction between cocatalysts (such as MAO) and support surfaces in supported catalysts is still insufficient.

Future development directions: (1) Develop in situ liquid/solid combined NMR technology to monitor the anchoring and activation of catalyst molecules in real time during loading and activation. (2) Use multinuclear NMR such as ^27^Al and ^29^Si combined with DNP enhancement to study the relationship between MAO distribution, aggregation state on silica surface and active center formation. (3) Carry out high-field ^91^Zr or ^47/49^Ti solid-state NMR to directly detect the coordination environment and symmetry changes in metal centers after loading. (4) Combine DFT calculations and periodic models to establish the correlation between supported catalyst structure and NMR parameters.

## 3. Homogeneous Molecular Catalysts

### 3.1. Molecular Structure of Catalyst

#### 3.1.1. Metallocene Catalysts

**Figure 5:**
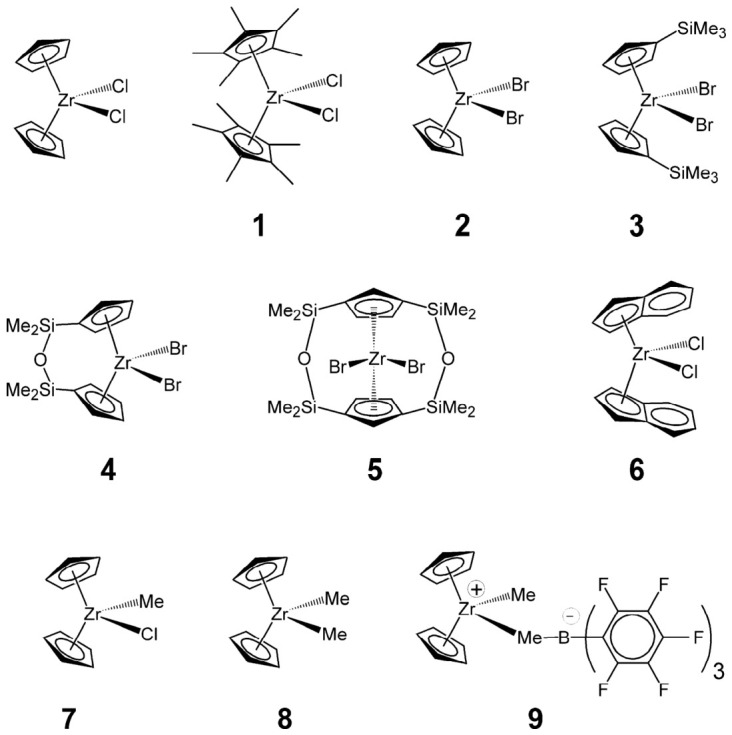
Representative metallocene catalysts. Reprinted with permission from [69]. (Bis(pentamethylcyclopentadienyl)zirconium Dichloride [Cp*_2_ZrCl_2_ (**1**)], Bis(cyclopentadienyl)zirconium Dibromide [Cp_2_ZrBr_2_ (**2**)], Bis(trimethylsilylcyclopentadienyl)zirconium Dibromide [(Me_3_SiC_5_H_4_)_2_ZrBr_2_ (**3**)], [O(Me_2_SiC_5_H_4_)_2_ZrBr_2_ (**4**)], [(1,3-C_5_H_3_)(SiMe_2_OSiMe_2_)_2_(1,3-C_5_H_3_)ZrBr_2_ (**5**)], Bis(indenyl)zirconium Dichloride [Ind_2_ZrCl_2_ (**6**)], Bis(cyclopentadienyl)methylzirconium Chloride [Cp_2_ZrMeCl (**7**)], Bis(cyclopentadienyl)dimethylzirconium [Cp_2_ZrMe_2_ (**8**)], and the Active Polymerization Catalyst [Cp_2_ZrMe][MeB(C_6_F_5_)_3_] (**9**)).

Metallocene catalysts refer to catalysts composed of metal cations and cyclopentadienyl (Cp) or its derivative (indenyl, fluorenyl) anions through η-type coordination. They can have two Cp ligands (biscyclopentadienyl metal) or only one (monocyclopentadienyl metal). The most common ones for olefin polymerization are group IV elements (Ti, Zr, Hf) metallocene compounds. Figure 5 presents several typical metallocene catalysts with Zr as the metal center.

**Figure 6:**
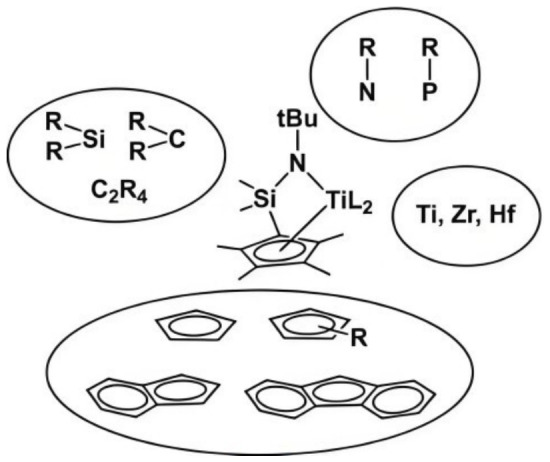
Structural diversity of group IV metal-based CGCs. Reprinted with permission from [73].

Constrained geometry complexes (CGCs) are a special type of monometallocene compounds whose ligands usually consist of a Cp group and an amine side arm [70,71,72] (shown as Figure 6). Compared with conventional biscyclopentadienyl metal catalysts, CGCs show significantly higher copolymerization ability in ethylene/long-chain α-olefin copolymerization.

Dibdalli et al. [74] studied the molecular structure, configurational isomers and ligand chemical environment of synthesized binuclear zirconium metallocene complexes using ^1^H and ^13^C NMR spectroscopy. Experimental results reveal that the complex exists exclusively as a single anti-configurational isomer. This originates from the steric hindrance of the CpZrCl_2_ fragment, which selectively prevents the attachment of a second metal moiety on the same side of the asymmetric indenyl-bridged ligand. Specifically, the ^1^H NMR spectrum (Figure 2 on page 3 in Dibdalli et al.’s work [74]) enables the clear assignment of all proton signals. Methyl protons on the pentamethylcyclopentadienyl (Cp) ligand appear at 2.02 ppm; proton signals located on the five-membered ring of the asymmetric indene framework are observed in the range of 6.20–6.33 ppm, while the resonances of 4 and 5 position protons on the asymmetric indene skeleton occur at 7.27 ppm. The ^13^C NMR spectrum (Figure S1 in Supporting Information of Dibdalli et al.’s work [74]) further corroborates the molecular structure. The methyl carbon signal of the Cp ligand is detected at 12.66 ppm, and the quaternary carbon signals of the bridged ligand and Cp* are concentrated within 124–127 ppm. Collectively, these spectroscopic data confirm the successful synthesis of the target complex and verify its well-defined molecular structure.

#### 3.1.2. Post-Metallocene Catalysts

Many new types of ligands have emerged after Cp ligands, with classic ligands including ONNO type (phenol imine), O4 type (tetradentate O4 ligand with phenol and ether donors), NN type (diamine, pyridine amine), NPN type (phosphine amine), etc. Catalysts composed of group IV metals and these new ligands are usually called post-metallocene catalysts.

**Figure 7:**
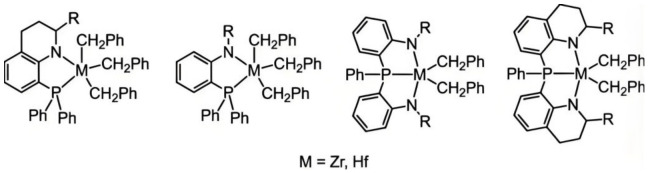
Phosphine-amide-ligated zirconium and hafnium catalysts. Reprinted with permission from [75].

Group IV metal complexes containing phosphine/amide mixed-donor ligands can also be used for ethylene polymerization. Toda et al. [75] reported zirconium complexes [NPN]Zr(NEt_2_)_2_ carrying [NPN] tridentate ligands, which can be used for PE synthesis. A variety of zirconium and hafnium complexes bearing amide-phosphine-type ligands ([PN] or [NPN]) have been reported in the literature (Figure 7) [76,77].

It is generally recognized that post-metallocene catalysts require ligands with appropriate rigidity and precisely oriented donor sites. For instance, five-membered chelating rings usually exhibit the highest stability, followed by six-membered rings. A similar stability trend can also be observed in sulfur- or oxygen-containing ligands, which are commonly bridged by an ortho-phenylene group [78,79,80,81,82,83]. The introduction of a methylene spacer between the aromatic ring and sulfur atom can effectively increase molecular flexibility [78,79].

**Figure 8:**
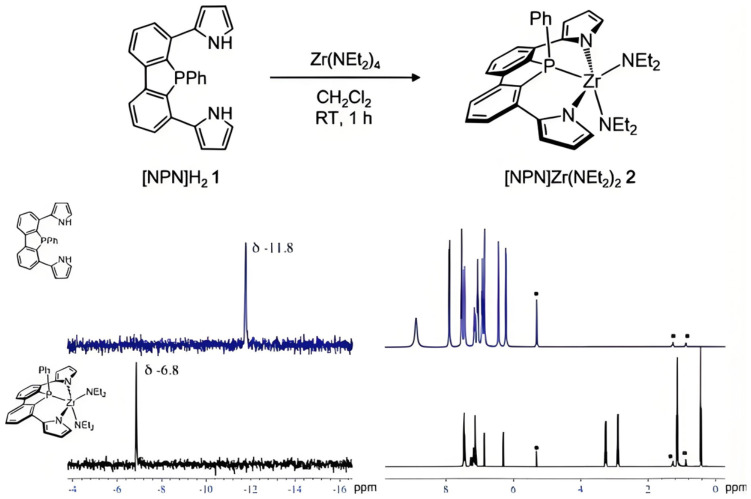
Synthesis of compound **2** and the ^31^P and ^1^H NMR spectra of compounds **1** and **2**. Reprinted with permission from [75].

In the ^1^H NMR spectrum of compound **2**, the pyrrole N-H signal (2H) disappears at 8.9 ppm (Figure 8), and the spectrum shows no significant change when the measurement temperature rises from room temperature to 70 °C, indicating that the [NPN] ligand binds tightly to the zirconium center. Compound **2** exhibits no obvious dynamic behavior on the NMR timescale.

In the ^31^P NMR spectrum, a downfield shift is observed from compound **1** to compound **2** (δ_P_ = −11.8 to −6.8, Figure 8). It has been reported that zirconium amide complexes with analogous [NPN] ligands also display a similar downfield variation in ^31^P chemical shifts [76,77].

**Figure 9:**
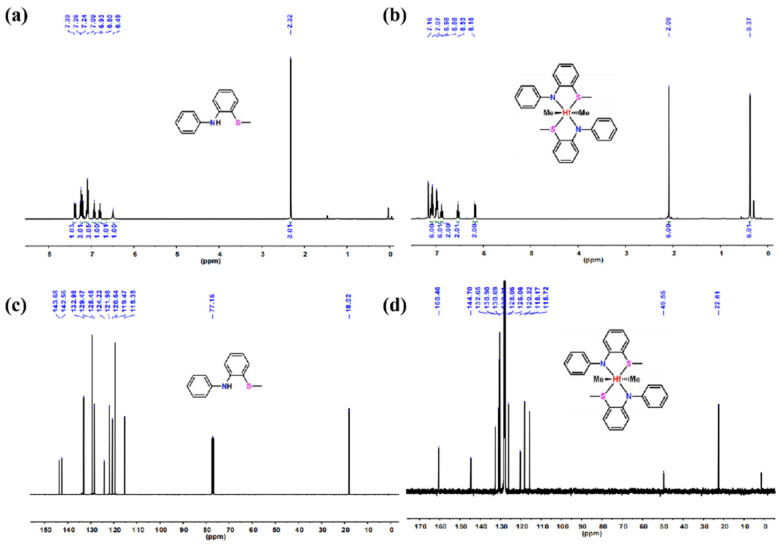
(**a**) ^1^H NMR spectrum of ligand L in CDCl_3_; (**b**) ^1^H NMR spectrum of the Hf complex in C_6_D_6_; (**c**) ^13^C NMR spectrum of ligand L; (**d**) ^13^C NMR spectrum of the Hf complex. Reprinted with permission from [84].

The ligand L displayed in Figure 9 contains amide and thioether functional groups. In general, metal coordination reduces the electron density around these groups, thereby inducing a de-shielding effect and resulting in a downfield shift in the corresponding hydrogen atoms (an increase in chemical shift δ). However, no obvious increase in δ was observed between Figure 9a,b. This discrepancy may stem from the use of different solvents: the ^1^H NMR spectrum of the Hf complex was measured in C_6_D_6_, while the NMR spectrum of the free ligand was recorded in CDCl_3_.

Owing to π–π interactions or anisotropic effects, C_6_D_6_ exerts a pronounced solvent effect on hydrogen atoms adjacent to polar groups or aromatic rings, which enhances shielding and leads to an up-field shift (a decrease in δ). Consequently, the de-shielding effect caused by metal coordination may be partially counteracted or masked by solvent influences.

Further comparison between Figure 9c,d reveals a significant downfield shift in the signal region of aromatic carbon atoms near the amide nitrogen (N-Aryl C, approximately 140–160 ppm). Since these carbon atoms are directly bonded to the coordinated nitrogen center, coordination decreases the electron density of nitrogen. Through the inductive effect, the electron deficiency of the connected aromatic carbons is enhanced, producing a de-shielding effect and ultimately causing a downfield shift in these aromatic carbon signals. These results confirm the successful coordination between Hf and the ligand and the formation of the target precatalyst.

#### 3.1.3. Late Transition Metal Catalysts

Late transition metal catalysts refer to catalysts centered on late transition metals (Ni, Pd, Fe, Co, etc.). They have unique catalytic behaviors, such as α-diimine nickel catalysts often having high chain-walking frequency to produce hyperbranched polyolefin structures, and high tolerance for polar functional group-containing monomers [85].

Antonov et al. [86] synthesized a series of Ni(II) dichloro complexes containing bis(imine)pyridine ligands with electron-withdrawing groups on the benzene ring (Table 1). The ^1^H NMR spectra of these Ni(II) complexes show obvious paramagnetic shifts, indicating that the d^8^ Ni(II) center is in a high-spin state (S = 1).

In the case of late-transition metal catalysts containing nickel, some systems exhibit paramagnetic behavior, while others are diamagnetic. To date, no researchers have established a practical criterion for determining whether a nickel-centered complex is paramagnetic or diamagnetic in the absence of NMR measurements. We have conducted in-depth consideration of this question, and propose that the key lies in analyzing the number of unpaired electrons at the metal center.

According to Crystal Field Theory, the metal–ligand interaction in a coordination complex is primarily electrostatic, similar to the cation–anion interaction in an ionic crystal. Under the influence of the surrounding ligand field, the five originally degenerate d orbitals of the central metal ion split into discrete energy levels. This orbital splitting induces electron redistribution within the d orbitals, thus stabilizing the system. The specific electronic configuration, whether high-spin or low-spin, is determined by the relative magnitudes of the orbital-splitting energy (Δ) and the electron-pairing energy (P).

The first step requires determining the oxidation state of the central ion, which defines the number of d electrons. The most common oxidation state is +2 (corresponding to a d^8^ configuration), although species such as Ni^+^ (d^9^) and Ni^3+^ (d^7^) are also accessible. Subsequently, the geometric configuration of the complex must be identified to predict whether the electrons adopt a high-spin (with unpaired electrons, except for d^10^) or low-spin (with all electrons paired, for even d-electron count) state. The spin state depends on multiple factors, including the property of the ligands and the electronic structure of the nickel ion. For the most common Ni(II) d^8^ system, its magnetic properties are geometry-dependent. A square-planar geometry, typically stabilized by strong-field ligands, results in a low-spin electron configuration with all electrons paired, thereby exhibiting diamagnetism. Conversely, a tetrahedral geometry, often formed with weak-field ligands, adopts a high-spin configuration featuring two unpaired electrons, which confers paramagnetism. The octahedral geometry is invariably paramagnetic due to the presence of two unpaired electrons, as only one electron configuration exists for this geometry. The ligand field strength is directly correlated with the resulting geometry: strong-field ligands favor the diamagnetic, square-planar structure by significantly increasing the splitting energy (Δ), thereby showing preference to the low-spin configuration. In contrast, weak-field ligands tend to form either tetrahedral or octahedral geometries, which are paramagnetic. Regarding other oxidation states, both Ni^+^ (d^9^) and Ni^3+^ (d^7^) invariably exhibit paramagnetism due to the presence of at least one unpaired electron in their electronic configurations [87].

The spin state, either high or low, is not fixed. When Δ ≈ P, the spin state becomes labile, allowing for spin-crossover behavior induced by external factors (e.g., temperature, solvent).

#### 3.1.4. Summary and Outlook

At present, for catalysts containing paramagnetic metal centers (such as Ni(II), Fe(II)), NMR signals are severely broadened, making it difficult for conventional NMR to obtain high-resolution structural information. The correlation between dynamic conformation, hindered rotation behavior and catalytic performance of ligands in solution is not thoroughly studied. Quantitative NMR characterization of ligand electronic and steric effects in late transition metal catalysts is lacking.

Future development directions: (1) Develop paramagnetic NMR technology (such as ^1^H NMR paramagnetic shift analysis, T_1_ measurement) to characterize the ligand structure and spin state of catalysts such as Ni(II) and Fe(II). (2) Use ^19^F NMR and HOESY techniques to detect weak interactions between fluorine-containing ligands and catalyst active centers and their effects on catalytic performance. (3) Combine variable-temperature NMR and DOSY to study the conformational change, kinetic processes and aggregation behavior of catalysts in solution. (4) Carry out DFT-assisted NMR parameter calculations to establish relationships between ligand structure, metal electronic environment and NMR spectra.

### 3.2. Catalyst/Cocatalyst Interactions

#### 3.2.1. Types of Cocatalysts

In olefin coordination polymerization, single-component organometallic compounds are usually called precatalysts, which need to be activated by cocatalysts to generate real active species [84,88,89]. Common cocatalysts mainly include aluminum alkyls, methylaluminoxanes, organoboranes and organoborates. Typical aluminum alkyls cover trimethylaluminum (TMA), triethylaluminum, triisobutylaluminum, trihexylaluminum, trioctylaluminum, diethylaluminum chloride, and ethylaluminum dichloride, among others. Common aluminoxanes consist of methylaluminoxane (MAO) and modified methylaluminoxane (MMAO). The representative organoborane is tris(pentafluorophenyl)borane. Organoborates refer to a class of salts with tetrakis(pentafluorophenyl)borate as the anion. Their cations are generally strongly acidic groups, which can cationize alkylated olefin polymerization catalysts.

**Figure 10:**
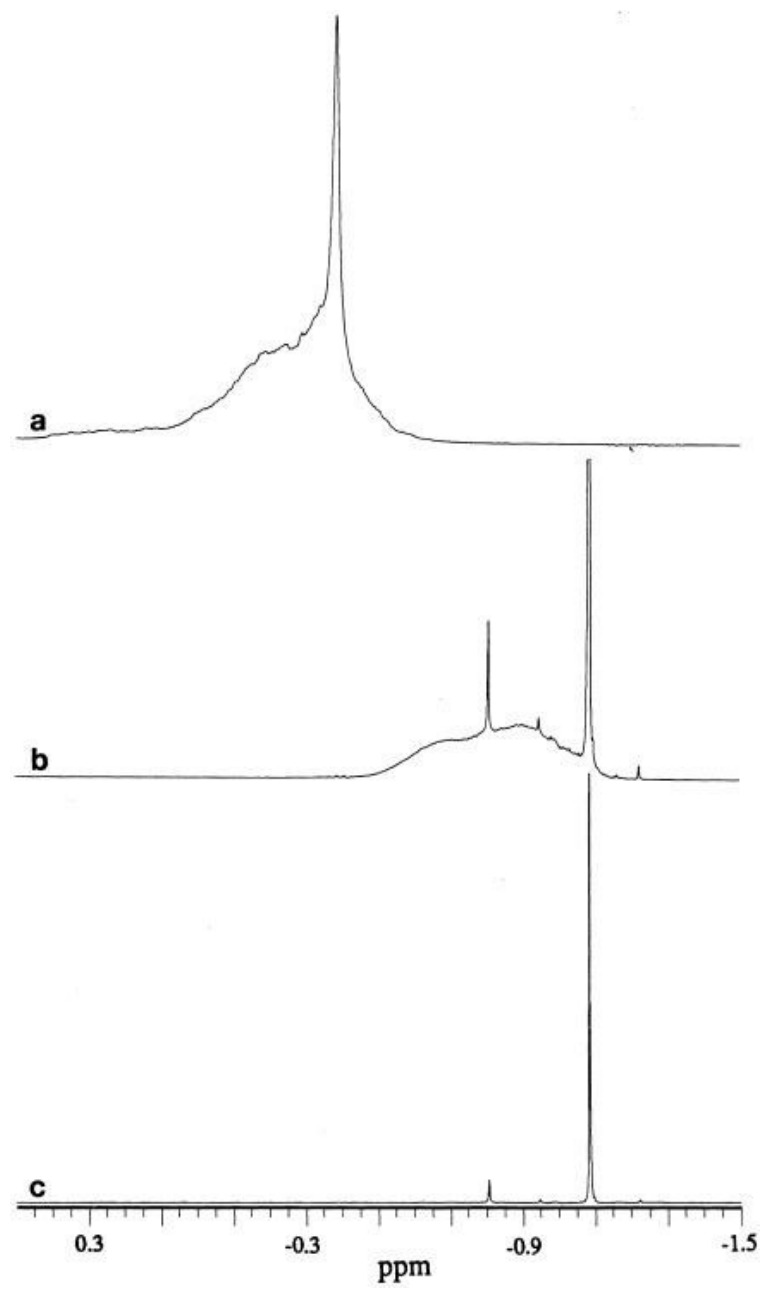
Proton NMR spectra of 30% MAO in toluene (400 MHz; 0.5 to −1.5 ppm). (**a**) Sample diluted in perdeu-teriobenzene only. The TMA peak is at −0.37 ppm, while the broader feature, 0.5 to −0.7 ppm, is the MAO. (**b**) Sample diluted in four volumes of THF-d8. The TMA peak is at −1.08 ppm. This spectrum is expanded vertically to show the broad MAO peak, −0.3 to −1.3 ppm. (**c**) Spectrum b with the MAO removed via curve fitting. The small peak at −0.8 ppm is a low-molecular-weight species or end group from the MAO. The T_1_ values of this peak closely matches the MAO and is much shorter than that of TMA. Reprinted with permission from [90].

The structure of alkylaluminoxane is unclear, usually prepared by controlled hydrolysis of alkyl aluminum. Taking MAO as an example, it is generated by hydrolysis of TMA, with complex structure and often difficult to define clearly, usually expressed as [Al(μ-O)Me]_n_. Imhoff et al. [90] added excess deuterated tetrahydrofuran (THF-d_8_) to a toluene solution of MAO, which shifted and significantly narrowed the ^1^H NMR peaks of TMA, thereby achieving effective peak separation from the broad signals of MAO. After eliminating residual peak overlap via curve fitting, the TMA content could be accurately quantified (Figure 10). Combined with independent determination of total aluminum content, the average number of methyl groups bonded to each aluminum atom in MAO was calculated. This method enables rapid and accurate measurement of TMA content. The results revealed that each aluminum atom in MAO is bound to an average of 1.4–1.5 methyl groups, with an oxygen-to-aluminum ratio of 0.75–0.80, revising the traditional assumption of a 1:1:1 stoichiometric structure.

**Figure 11:**
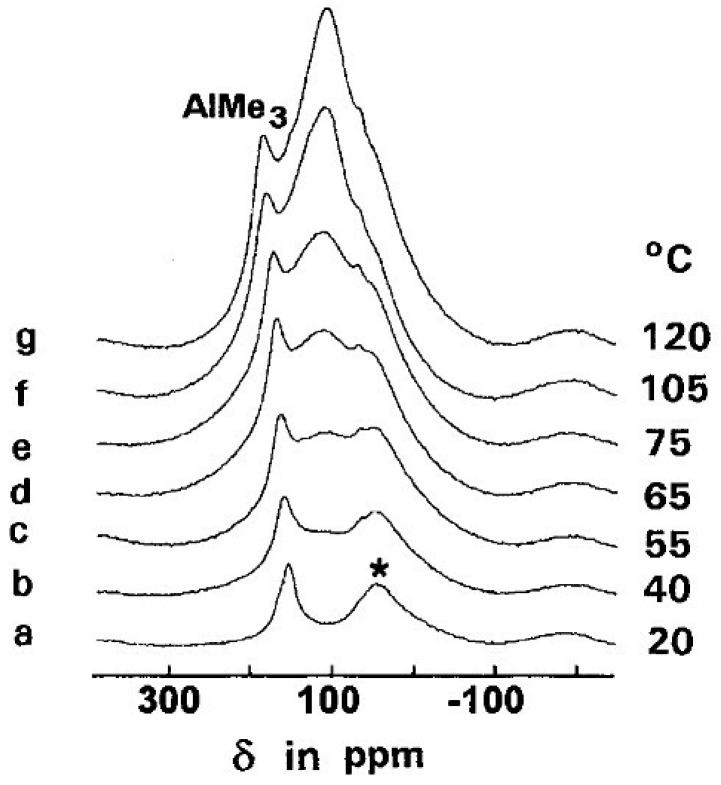
^27^Al NMR spectra of MAO-2 solution in toluene (2.0 M) at various temperatures: 20 °C (**a**); 40 °C (**b**); 55 °C (**c**); 65 °C (**d**); 75 °C (**e**); 105 °C (**f**); 120 °C (**g**). Asterisk (*) marks the background signal of NMR probe head. Reprinted with permission from [91].

Babushkin et al. [91] systematically investigated the multinuclear NMR behavior of MAO in toluene solution. It was found that the ^27^Al NMR signal of MAO is extremely broad at room temperature (δ ≈ 60 ppm, line width of approximately 50 kHz) and difficult to observe under conventional conditions. When the temperature was increased to 40–120 °C, a signal appeared at δ ≈ 110 ppm with a line width of 10–15 kHz, which was consistent with the chemical shift in known cage-shaped tert-butylaluminoxane clusters (Figure 11).

Activation of precatalysts by MAO usually undergoes two steps: (1) Ligand exchange: replacing halogen ligands with alkyl groups (e.g., Me, i-Bu) to form metal–carbon bonds as initiation sites for chain growth; (2) Ionization: abstracting an alkyl anion from the alkylated metal center to generate cationic active species [L_n_Mt-R]^+^, accompanied by the formation of corresponding [MAO-R]^−^ anions [92,93].

Rocchigiani et al. [94] found that the reaction of the precatalyst Cp_2_ZrMe_2_ with MAO (or DMAO) undergoes alkyl exchange and anion capture to generate two categories of ion pairs [95,96,97], namely an outer-sphere ion pair (OSIP 1) and inner-sphere ion pair (ISIP 2) [98]. Their structural diagrams are presented in Scheme 1 on page 1 of Rocchigiani et al.’s work [94]. It depicts the formation pathways of OSIP 1 and ISIP 2 from the reaction between Cp_2_ZrMe_2_ and excess MAO.

The feature of OSIP 1 is that the anion [MeMAO]^−^ resides in the outer coordination sphere of the zirconium cation. Zr and Al are linked by two methyl bridges (μ-Me), affording a loosely packed and spatially extended ion-pair structure. In contrast, in ISIP 2, [MeMAO]^−^ closely interacts with the Zr center through a single methyl group and is located in the inner coordination sphere. This structure is more compact and approximates the state of “free ions” [99,100,101,102].

The second type of cocatalysts are borate-based or “non-coordinating anion” activators, representing a new generation of high-efficiency activators. A typical example is [Ph_3_C][B(C_6_F_5_)_4_], which consists of a bulky organic cation (Ph_3_C^+^) and a weakly coordinating anion B(C_6_F_5_)_4_^−^. Its activation mechanism involves abstracting methyl groups (or other alkyl groups) from the metal center to form cationic active metal species. The B(C_6_F_5_)_4_^−^ anion features a large steric volume and strong electron-withdrawing ability, and can hardly coordinate strongly with the metal center. This maintains the Lewis acidity of the metal center and facilitates olefin insertion [88]. Unlike MAO, borate activators exhibit weak scavenging capacity. Therefore, their application requires strict anhydrous and oxygen-free experimental conditions. Their key advantage is the absence of additional aluminum species, which effectively avoids aluminum-related side reactions.

In general, borate activators possess distinctly stronger ionization capacity than MAO. They can generate active centers more rapidly and quantitatively, and deliver higher initial catalytic activity under identical conditions. Nevertheless, aluminum species in MAO systems can stabilize active centers to a certain extent and suppress their decomposition. In contrast, pure borate systems lack such stabilization effects, resulting in a shorter lifetime of active centers and faster activity attenuation. Furthermore, the powerful impurity-scavenging performance of MAO endows it with higher tolerance to trace impurities, making it more robust for industrial applications [21,25,38].

#### 3.2.2. Mechanism of Cocatalysts

**Figure 12:**
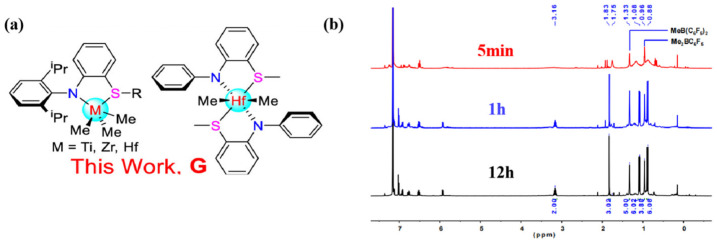
(**a**) Metal complexes containing soft sulfur donor(s); (**b**) Stacked ^1^H NMR spectra of the Hf system in C_6_D_6_ at different times after the addition of one equivalent of B(C_6_F_5_)_3_. Reprinted with permission from [84].

The triphenylmethyl cation ([Ph_3_C]^+^) is a strong Lewis acid that can efficiently abstract methyl anions (CH_3_^−^). The cationic active species formed after abstraction forms loose ion pairs with [B(C_6_F_5_)_4_]^−^, stabilizing the cations without deactivation caused by strong coordination. Li et al. [84] adopted group IV metal (Hf, Zr, Ti) methyl complexes as precatalysts, with the general formulas L_2_M(CH_3_)_2_ and LM(CH_3_)_3_, where L denotes a thioether-amide bidentate ligand (see Figure 12a for its structure).

The mechanism proposed in this article is as follows: [Ph_3_C]^+^ first attacks and abstracts one methyl group (CH_3_^−^) from the precatalyst, producing triphenylmethane (Ph_3_C–CH_3_) as a by-product. The removal of the methyl anion converts the neutral Hf center into a monocationic center with reduced electron count and coordination number, generating a coordinatively unsaturated site. The resulting [(L)Hf(CH_3_)_2_]^+^ subsequently forms an ion pair with the bulky [B(C_6_F_5_)_4_]^−^. This process can be briefly summarized as:


(L)Hf(CH_3_)_3_ + [Ph_3_C][B(C_6_F_5_)_4_] → [(L)Hf(CH_3_)_2_]^+^[B(C_6_F_5_)_4_]^−^ + Ph_3_C–CH_3_

Rocchigiani et al. [94] demonstrated that the reaction of the precatalyst Cp_2_ZrMe_2_ with MAO/DMAO yields two ion-pair species: OSIP ([Cp_2_Zr(μ-Me)_2_AlMe_2_]^+^ MeMAO^−^) and ISIP ([Cp_2_Zr–Me···MeMAO]^+^). The core step of this activation process is that MAO/DMAO acts as a Lewis acid to abstract alkyl groups, thereby generating metallocene zirconium cationic centers ([Cp_2_Zr–R]^+^) and anionic moieties (MeMAO^−^), which subsequently form stable ion pairs through electrostatic interactions. The authors quantitatively compared the aggregation behaviors of different systems via diffusion NMR, providing direct evidence that the formation of ion pairs serves as the dominant mechanism for the activation effect of MAO cocatalysts.

#### 3.2.3. In Situ NMR Studies

Li et al. [84] simulated and monitored the chemical interaction between B(C_6_F_5_)_3_ and the Hf precatalyst via in situ NMR experiments. The precatalyst was mixed with B(C_6_F_5_)_3_ in C_6_D_6_, and ^1^H and ^11^B NMR spectra were recorded at 5 min, 1 h, and 12 h, respectively. As shown in Figure 12b, two new methyl signals appeared at 1.33 ppm and 0.96 ppm in the spectra, which were assigned to MeB(C_6_F_5_)_2_ and Me_2_BC_6_F_5_, respectively. These results indicate that B(C_6_F_5_)_3_ does not directly abstract a methyl group to form the expected MeB(C_6_F_5_)_3_^−^. Instead, the transfer of C_6_F_5_ groups to the strongly electrophilic Hf center occurs.

Proposed reaction mechanism:

(L)HfMe_2_ + B(C_6_F_5_)_3_ → (L)Hf(C_6_F_5_)Me + MeB(C_6_F_5_)_2_

The presence of Me_2_B(C_6_F_5_) suggests that further decomposition or disproportionation reactions may occur, such as:

2 MeB(C_6_F_5_)_2_ ⇌ Me_2_BC_6_F_5_ + B(C_6_F_5_)_3_

**Figure 13:**
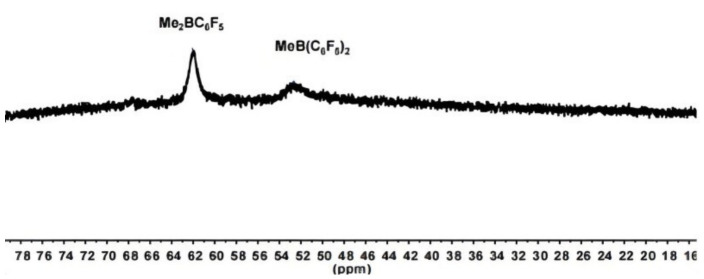
^11^B NMR spectrum of the Hf system in C_6_D_6_ after reacting with one equivalent of B(C_6_F_5_)_3_ for 12 h. Reprinted with permission from [84].

Figure 13 reveals the emergence of new signals distinct from those of the initial reactant, B(C_6_F_5_)_3_. These new signals are assigned to the decomposition products, MeB(C_6_F_5_)_2_ and Me_2_BC_6_F_5_.

In metallocene catalytic systems, the reaction between Cp_2_ZrMe_2_ and MAO does not produce a single active species, but rather establishes an equilibrium system containing multiple ion pairs, among which OSIP 1 and ISIP 2 are two critical types of species [27]. During chain propagation, olefin monomers need to approach the zirconium center and insert into the Zr-polymer chain bond. The structure of ISIP 2 is more conducive to monomer access to the Zr center, so it is regarded as a “monomer-accessible” species and thus exhibits high catalytic activity. In contrast, in OSIP 1, the zirconium center is shielded by bimetallic bridges and bulky anions, which greatly hinders monomer approaching. Therefore, OSIP 1 is defined as a “monomer-inaccessible” species with low or even negligible activity. Consequently, the overall polymerization activity of the system is directly determined by the ratio of OSIP 1 to ISIP 2 and their dynamic equilibrium.

The relationship between the aggregation state and kinetic behavior of ion pairs was quantitatively revealed by measuring the hydrodynamic volume (V_H_) and aggregation number (N). The research found that neither MAO nor DMAO exist as discrete small “cage-like” structures in solution. Instead, they self-aggregate to form large supramolecular structures, whose size increases with rising concentration. DMAO (MAO depleted of free AlMe_3_) exhibits a stronger tendency for self-aggregation and a larger volume than MAO. This indicates that the presence of AlMe_3_ suppresses the aggregation of MAO.

Upon the formation of ion pairs, their size and aggregation behavior are closely linked to MAO/DMAO. As shown in Figure 6 of Rocchigiani et al.’s work [94], the V_H_ of ISIP 2 as a function of concentration almost completely overlaps with the curve for DMAO. Furthermore, its aggregation number remains close to unity (N ≈ 1, ranging from 0.79 to 0.93), demonstrating that ISIP 2 possesses no intrinsic driving force for self-aggregation. In contrast, OSIP 1 exhibits a much steeper increase in V_H_ with concentration compared to ISIP 2 and DMAO, showing strong concentration-dependent aggregation (N increases from 0.96 to 1.63). This indicates that, beyond inheriting the aggregation from DMAO, OSIP 1 possesses a significant molecular dipole moment, which provides an additional, strong driving force for its self-aggregation. The aggregation of multiple OSIP 1 entities into larger superstructures further shields the active centers, significantly worsening their kinetic accessibility and consequently severely inhibiting polymerization activity.

#### 3.2.4. Balance Between Sensitivity and Signal Resolution

Gao et al. [88] indicate that the reaction of oxazoline-amine zirconium complex precatalysts with the cocatalyst Ph_3_C^+^·B(C_6_F_5_)_4_^−^ produces cationic active species, along with the byproduct Ph_3_C–CH_2_Ph, which forms from the combination of triphenylmethyl cations and benzyl groups. This results in complex system components, severe signal overlap, and difficult NMR spectral assignment. To solve this problem, the authors introduced 1,2-difluorobenzene (DFB) into the NMR samples. The fluorine atoms in DFB have high electronegativity, and its benzene ring presents magnetic anisotropy, allowing weak interactions with cationic species in solution. This noticeably changes the proton chemical shifts in cationic species (typically inducing downfield shifts) and moves target signals away from the overlapping region. For example, after the addition of DFB, the benzyl proton signals of the active species L-Zr^+^(CH_2_Ph) exhibit a distinct downfield shift and separate from the byproduct signals (Figure S35 in Gao et al.’s work [88]), thereby providing ^1^H NMR spectra with higher resolution. This strategy effectively balances sensitivity and resolution in complex systems.

#### 3.2.5. Summary and Outlook

At present, for cocatalysts with unclear structures such as MAO, their real structures and activation mechanisms in solution are still controversial, and NMR studies are mostly limited to qualitative or semi-quantitative ones. The quantitative correlation between ion pair structure dynamic equilibrium, aggregation behavior and polymerization activity is insufficient. NMR studies on the synergistic effect of borate activators and alkyl aluminum are few.

Future development directions: (1) Carry out multinuclear (^1^H, ^13^C, ^27^Al, ^11^B) in situ NMR to monitor the activation process of cocatalysts and precatalysts in real time under reaction conditions. (2) Use diffusion NMR (DOSY) and NOE/ROE techniques to study the relationship between ion pair aggregation state, hydrodynamic volume and active center accessibility. (3) Combine DFT calculations to predict ion pair structures and NMR parameters. (4) Develop DNP enhancement technology to improve detection ability for low-concentration, short-lived intermediates.

### 3.3. Catalyst/Cocatalyst/Monomer Interactions

In situ NMR technology enables the real-time monitoring of dynamic processes in homogeneous catalyst/cocatalyst/monomer systems under actual reaction conditions, providing critical molecular-level evidence for elucidating the formation mechanism of catalytic active sites, the activation role of cocatalysts, and the kinetics of monomer insertion and chain propagation. This technique captures the evolution of transient intermediates, tracks chemical shift changes close to metal centers, and quantitatively analyzes monomer consumption and polymer formation behavior, thereby clarifying the structural origins and regulatory mechanisms of stereoselectivity. By integrating multi-nuclear and multi-dimensional spectral analysis with isotope labeling strategies, in situ NMR offers irreplaceable advantages in resolving the synergistic effects of ligand fine-tuning, metal electronic properties, and spatial environments on polymer stereoregularity. It thus serves as a crucial bridge connecting static catalyst structures with dynamic polymerization behaviors.

#### 3.3.1. Monomer Insertion Mechanism in Late Transition Metal Catalysts

α-diimine Ni(II) and Pd(II) complexes are highlighted as a groundbreaking class of late-transition metal catalysts [103,104]. Their key feature are the bulky aryl substituents on the diimine ligand (Figure 1 in Pellecchia et al.’s work [85]), which suppress chain transfer and enable formation of high-molecular-weight polymers through polymerization instead of oligomerization [103].

A defining feature is their unique “chain-walking” mechanism, which represents a pivotal departure from conventional early-transition metal catalysis: following monomer insertions, a series of rapid and reversible β-hydride eliminations and reinsertions allows the metal center to migrate along the growing polymer chain before subsequent monomer insertion occurs. This process, proceeding through a cationic alkyl ethylene complex and a β-agostic intermediate, enables a single ethylene monomer to generate branches, resulting in the formation of highly branched PE directly from ethylene feedstock without comonomers [37,38]. The degree of branching and the final polymer architecture are highly dependent on the metal (Ni vs. Pd), the steric bulk of the diimine ligand, and the reaction conditions.

Through the use of a ^13^C-enriched catalyst precursor, Ni–^13^CH_3_, Pellecchia et al. [39] investigated the stereochemistry of the chain initiation step in the propylene polymerization mediated by α-diimine nickel catalysts. The experimental results revealed that only two of the four possible diastereomers were detected, with comparable intensities, indicating that stereocontrol becomes operative upon the formation of the first chiral carbon in the growing chain. In contrast, for vanadium-based catalysts, all four possible diastereomeric [7–^13^C]-2,4,6-trimethylheptyl end groups were observed (Scheme 4 in Pellecchia et al.’s work) [85], demonstrating that the primary insertion of the monomer is stereoirregular during both the initiation and the first two propagation steps. This comparative analysis highlights the unique characteristics of this class of late-transition-metal catalysts.

#### 3.3.2. In Situ NMR Observation Examples

**Figure 14:**
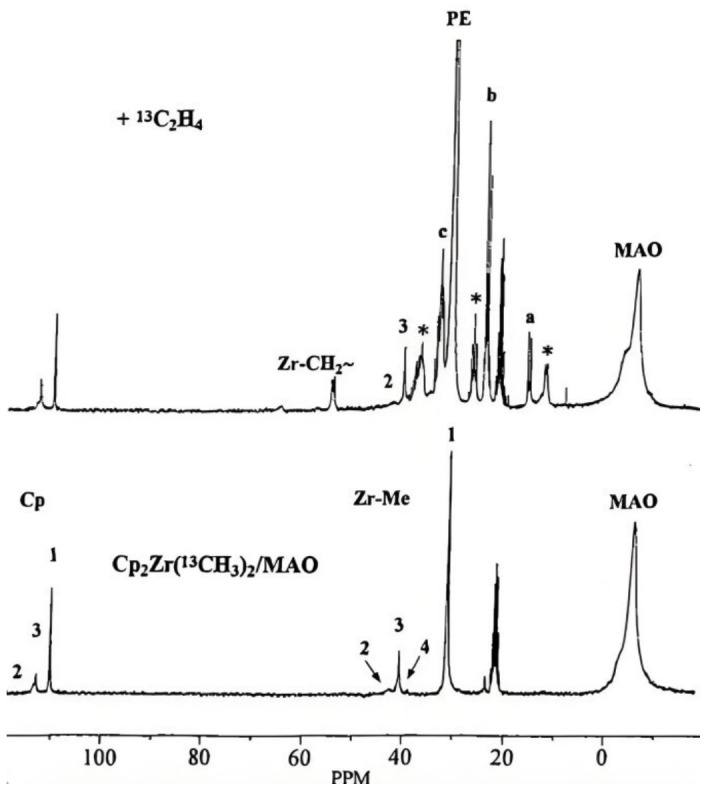
The ^13^C NMR spectra at −20 °C in toluene-d_8_ of Cp_2_Zr(^13^CH_3_)_2_ (90% ^13^C-enriched)/MAO (**bottom**) and Cp_2_Zr(^13^CH_3_)_2_/MAO/^13^C_2_H_4_ (90% ^13^C-enriched) (**top**). [Zr] = 0.07 mol·L^−1^, [Al]/[Zr] = 20, [^13^C_2_H_4_] = 0.4 mol·L^−1^. Peaks a, b, and c correspond to the multiplet signals of the terminal methyl (a), terminal methylene of the ethyl (b), and terminal methylene of the n-propyl (c) groups of the polyethylene chain, respectively. The asterisks (*) indicate the signals of the methylene carbons at the α (d, 11.24 ppm), β (m, 25.83 ppm), and γ (m, 36.40 ppm) positions of the polymer chain bound to aluminum. Reprinted with permission from [41].

In a groundbreaking study by Tritto et al. [41], in situ ^13^C NMR spectroscopy was employed to monitor the ethylene polymerization process at the molecular level in real time, leading to the first direct observation of zirconium-polymeryl (Zr-polymeryl) active species via in situ NMR. The key spectroscopic evidence for this breakthrough, as shown in the upper spectrum of Figure 14, was the appearance of a set of doublets in the chemical shift region of 55–65 ppm, with a measured ^13^C–^13^C spin–spin coupling constant (J_C–C_) of approximately 29.65 Hz.

The chemical shift value (δ ~55–65 ppm) is significantly downfield from the characteristic signal of the polyethylene backbone methylene groups (~30 ppm). This pronounced de-shielding effect unequivocally indicates that the carbon atom resides in an electron-deficient environment, which is a hallmark feature of being directly bonded (Zr–C) to a strong Lewis acidic, partially positively charged zirconium (Zr^2+^) center. Furthermore, the observation of a doublet—as opposed to a more complex multiplet—demonstrates that this methylene carbon (^13^CH_2_–) is coupled to only one other ^13^C nucleus. This spectral pattern is entirely consistent with the structure of the growing polymer chain terminus: Zr–^13^CH_2_–^13^CH_2_–P. The coupling constant of ~29.6 Hz is typical for a ^13^C–^13^C single-bond coupling in a methylene group, further confirming the presence of a carbon–carbon bond. It should be noted that the experiments were conducted at a low NMR field; it might be possible to observe doublet of doublets and complicated peaks with high-field NMR due to two and three bond couplings.

Together, these spectral features provide incontrovertible evidence that the signal originates from a Zr–^13^CH_2_–P species (Zr-polymeryl species), where P denotes the growing polyethylene chain. This constitutes the first direct spectroscopic observation of the active propagating chain end—the crucial intermediate formed after olefin insertion into the metal–carbon (Zr–C) bond—as postulated by the Cossee–Arlman mechanism.

**Figure 15:**
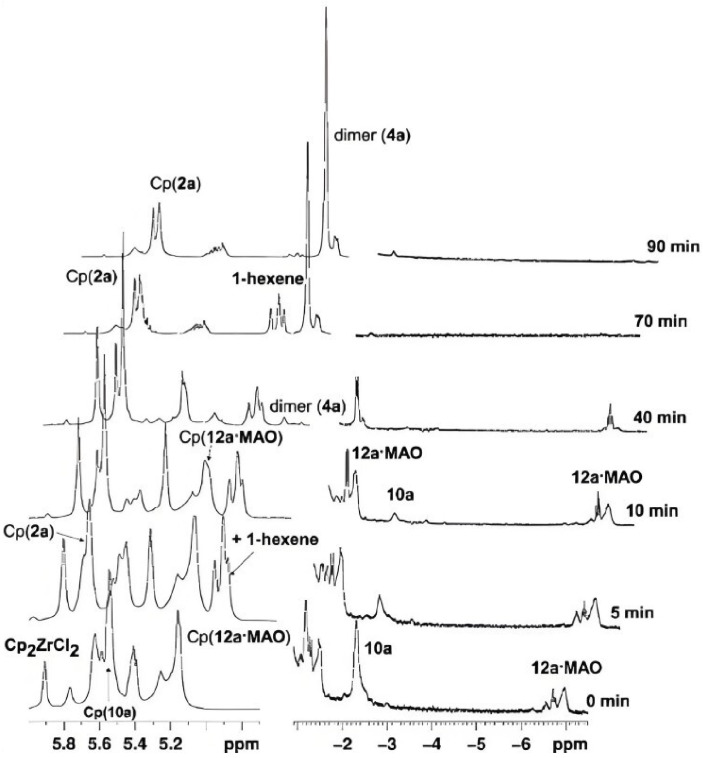
^1^H NMR monitoring of 1-Hexene transformation by the [Cp_2_ZrH_2_]_2_–ClAlMe_2_–MMAO-12 system in C_7_D_8_ (T = 26 °C, intensity of up-field signals magnified). Reprinted with permission from [105].

Parfenova et al. [105] visually demonstrated the time-dependent changes in key components in the catalytic reaction system composed of [Cp_2_ZrH_2_]_2_, ClAlMe_2_, and MMAO-12 with 1-hexene as the substrate using in situ ^1^H NMR spectroscopy (Figure 15). In the spectrum at the initial reaction time (*t* = 0 min), signals at −6.56 ppm (triplet, t) and −1.08 ppm (doublet, d) were observed. Simultaneously, the corresponding proton signal of the Cp rings appeared near 5.42 ppm. These signals represent the bimetallic zirconium hydride structure bound to MMAO (x[Cp_2_ZrH_2_·Cp_2_ZrHCl·ClAlR_2_]·yMAO), which is the catalytically active species. They serve as the key active centers for initiating and proceeding the alkene dimerization. As the reaction progressed from 0 to 60 min, the signals corresponding to the active species 12a-MAO (−6.56 ppm and −1.08 ppm) significantly weakened and eventually disappeared almost completely. This indicates that the active species was consumed during the reaction process, participating in the transformation of 1-hexene into products. The multiplet appearing in the range of 4.5–5.0 ppm corresponds to the proton signal of the vinylidene group (>C=CH_2_) in the dimer products. This is the characteristic signal for the selective “head-to-tail” dimerization of 1-hexene. This in situ monitoring provides the most direct experimental evidence that the bimetallic zirconium hydride complex is the true active center for alkene dimerization.

Gafurov et al. [106] systematically investigated the catalytic behavior of benzothiazole-based unsymmetric PCN pincer nickel(II) complexes, (BzTPCN)NiX (X = F, Br), in ethylene oligomerization (Figure S2 in Gafurov et al.’s work [106]). To elucidate the evolution of the catalyst during activation with MMAO, the authors employed ^31^P NMR spectroscopy as a key diagnostic tool. The experimental results revealed that the initial complexes (BzTPCN)NiBr and (BzTPCN)NiF in toluene solution exhibited sharp singlets in the ^31^P NMR spectrum at δp = 87.5 ppm and 83.3 ppm, respectively. Upon treatment with 400 equivalents of MMAO, the phosphorus signals for both complexes shifted up-field to δp = 81.2 ppm, while retaining their singlet shape. This distinct up-field shift confirms the substitution of the halide ligand (X^−^) by a methyl group, leading to the formation of a uniform methyl-nickel species, (BzTPCN)NiCH_3_. The result indicates that MMAO effectively promotes alkylation at the nickel center, generating a putative catalytically active precursor.

#### 3.3.3. Summary and Outlook

At present, in situ NMR studies mostly focus on model reactions (such as 1-hexene, ethylene). In addition, direct real-time NMR evidence for complex mechanisms such as chain-walking remains insufficient. Furthermore, in situ investigations on the dynamic evolution of metal–carbon bonds and stereochemical control during monomer insertion are still inadequate.

Future research directions are proposed as follows: (1) Develop high-pressure in situ NMR techniques to monitor monomer consumption, chain propagation and chain transfer under realistic polymerization pressures. (2) Employ helium-cooled cryoprobes to enhance measurement sensitivity and avoid reliance on ^13^C labeling, so as to track monomer insertion pathways and the structural evolution of chain ends. (3) Conduct variable-temperature in situ NMR experiments to investigate the activation energy and kinetics of chain-walking, *β*-hydride elimination and reinsertion processes. (4) Combine DFT calculations with kinetic simulations to establish quantitative models correlating NMR spectroscopic signals with polymerization mechanisms.

In situ NMR indeed holds significant research potential; however, its practical application faces considerable challenges. For instance, the reaction proceeds rapidly, requiring the detection of reaction processes within a very short time, which demands high sensitivity and resolution from the NMR equipment. Furthermore, the temperature and pressure conditions required for in situ olefin polymerization are stringent; to monitor the reaction process, the NMR instrument must be equipped with a cryogenic probe and a refrigeration unit. In addition, dissolution DNP represents another technique capable of enabling in situ NMR studies of olefin polymerization processes.

### 3.4. Stereochemical Control

**Figure 16:**
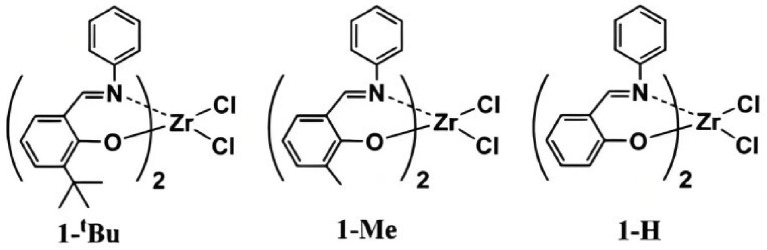
The zirconium complex studied. Reprinted with permission from [107].

Using NMR spectroscopy, Kravtsov et al. [107] thoroughly examined the relationship between the structures of intermediates and ethylene polymerization activities for three bis(phenoxyimino)zirconium catalysts with various substituents (1-^t^Bu, 1-Me and 1-H) activated by MAO and AlMe_3_/[CPh_3_]^+^[B(C_6_F_5_)_4_]^−^ (Figure 16).The findings showed that the type of activated intermediates and their catalytic performance are strongly influenced by the steric bulk of the substituent at the third position of the phenolic ring.

The bulky *tert*-butyl-substituted 1-^t^Bu, when activated with MAO, forms a highly active heterobinuclear ion pair [(L^tBu^)_2_Zr(μ-Me)AlMe_2_]^+^[Me–MAO]^−^, which exhibits significantly higher ethylene polymerization activity than the reference Cp_2_ZrCl_2_/MAO system. The smaller methyl-substituted 1-Me/MAO system, on the other hand, primarily produces a tight ion pair [(L^Me^)_2_ZrMe^+^···Me–MAO^−^], which significantly reduces polymerization activity. The unsubstituted 1-H completely breaks down into an inert aluminum ion pair [L^H^Al(μ–Me)(μ–Cl)AlMe_2_]^+^ during ethylene polymerization. Additionally, the coupling constants for the μ-Me and Al-Me groups in the heterobinuclear structure were clearly determined through ^13^C-labeling experiments, confirming the crucial role of steric effects in preventing tight coordination of the counteranion and maintaining the stability of the highly active intermediate. These discoveries offer crucial direction for the logical design of high-performance olefin polymerization catalysts by providing molecular-level insights into how ligand structure controls the activation pathways and performance of polymerization catalysts.

Razavi et al. [108] found that the stereoselectivity of the catalyst primarily originates from its inherent static geometric features, namely the bilateral symmetry and prochirality conferred by the ligand framework (such as the isopropylidene-bridged cyclopentadienyl-fluorenyl structure). These features determine the enantiomeric nature of the active sites, thereby establishing the structural foundation for producing stereoregular polymers with specific configurations (e.g., syndiotactic polypropylene).

However, the actual stereoregularity during polymerization is further governed by the dynamic behavior of the catalyst after activation, particularly the efficiency of the synergistic chain migration and active site isomerization. This dynamic process is profoundly influenced by the electronic properties of the central metal (such as the differences in Lewis acidity and ion-pairing tendencies between Hf and Zr caused by lanthanide contraction and relativistic effects) as well as ligand structural modifications (such as electron density redistribution induced by changes in the bridging groups, variations in ligand bonding modes, and alterations in frontier orbital energy levels and hybridization). The coupling of these electronic and steric factors ultimately precisely controls the stereochemistry of monomer insertion by modulating the propensity for α- or β-hydrogen interactions. Therefore, the stereoregularity of the polymer is not dominated by the properties of the monomer but is instead the result of a combination of the inherent static symmetry information encoded in the catalyst structure and the dynamic electronic and steric environment.

Most existing NMR studies focus on the static structure of catalysts, with a lack of real-time observation on the dynamic regulation mechanism of stereoselectivity during polymerization. There are limited investigations into the quantitative NMR correlation among ligand electronic effects, metal electronic properties and polymer stereoregularity. Moreover, in situ NMR evidence clarifying the influence of ion-pair structures on stereoselectivity is still scarce.

The prospects for future development are presented below: (1) Implement in situ ^13^C NMR with a cryoprobe to monitor the evolution polymers in real time during polymerization. (2) Combine DFT calculations to predict the effects of different ion-pair structures on the stereochemistry of monomer insertion, and further guide the structural optimization of catalysts. (3) Develop variable-temperature in situ NMR methods to explore the correlation between the dynamic equilibrium of ion pairs and stereoselectivity.

## 4. Conclusions and Outlook

In most cases, reaction mechanisms are primarily proposed based on theoretical speculation. In situ NMR can provide direct experimental evidence, thereby greatly improving the accuracy of mechanistic interpretation. In catalytic reactions, genuine active species are generally transformed from precatalysts. In situ NMR enables real-time monitoring of the activation process and identification of actual active centers. It occupies an irreplaceable position in accurately clarifying polymer chain structures and detecting key intermediates during chain propagation. Meanwhile, changes in chemical shifts can be used to deduce the coordination modes and interaction mechanisms between substrates and metal centers or catalyst functional groups. In polymer chemistry, material properties are ultimately determined by chain structure, sequence distribution, stereoregularity, and in situ NMR serves as a powerful tool for characterizing these critical parameters.

However, the application of in situ NMR to heterogeneous/homogeneous mixed systems faces a series of challenges, mainly arising from the physical property differences among components and the inherent limitations of NMR technology. These bottlenecks are expected to be gradually addressed through advanced methodologies. For instance, HR-MAS can significantly narrow spectral lines, render heterogeneous components “NMR-visible”, and distinguish interfacial species.

Signal overlap is an inherent limitation of NMR. NMR exhibits a relatively narrow range of chemical shift dispersion, especially for ^1^H NMR. Resonance signals of structurally similar yet functionally distinct molecules or functional groups appear at close chemical shifts, resulting in severe broad peak overlap and difficult peak assignment. Under in situ conditions, dynamic variations in temperature, pressure and concentration induce slight chemical shift drift, further broadening peak widths and shifting peak positions, and consequently exacerbating signal overlap.

In practice, HR-MAS addresses semi-solid and supported catalysts, DNP targets low-abundance surface species, in situ NMR captures real-time reaction dynamics, and machine learning streamlines spectral interpretation and high-throughput screening. Future research should focus on developing high-speed HR-MAS (>100 kHz) and DNP-enhanced NMR techniques to improve the detection capability for low-content and interfacial species. Additionally, with the rapid advancement of artificial intelligence, the integration of in situ NMR with computational modeling (such as DFT and machine learning) could become essential for predictive mechanistic analysis. Furthermore, artificial intelligence is expected to accelerate the innovation of advanced NMR technologies by improving spectral resolution and signal-to-noise ratios to resolve peak overlap (e.g., pure shift ^1^H NMR) and establish theoretical models to predict the structures of polymerization intermediates. Ye et al. [109] developed an AI-optimized quantitative RINEPT method combined with relaxation reagents, utilizing a simulated annealing algorithm to optimize the pulse sequence. This approach achieved a 7.5-fold improvement in sensitivity and reduced the single-scan acquisition time to 1/56 of that required by the original method, providing key technical support for high-throughput catalyst screening and the analysis of trace structural features such as long-chain branching. In parallel, Giampaolo et al. [110] developed an AI-powered automated analysis platform that integrates high-throughput experimentation with data science, enabling for the first time the automatic and rapid determination of the 13C NMR microstructure of polyolefins. This platform greatly reduces the time required for manual analysis and can be applied to the automated classification and microstructural characterization of polyolefin materials. Shortening experimental acquisition times will also promote broader application of in situ NMR. Multinuclear, multidimensional, variable-temperature and variable-pressure in situ NMR experiments should be systematically performed to establish a comprehensive molecular evolution map of catalytic processes. In summary, Phillips and Ziegler–Natta remain dominant for bulk polyolefins (HDPE), supported metallocenes excel in high-performance copolymers, late-transition metals enable polar monomer incorporation and novel topologies, while homogeneous metallocenes are most suitable for in-depth mechanistic elucidation. NMR is progressively shifting from static characterization toward dynamic, in situ, multinuclear, and quantitative analysis, which will unlock further potential for each catalyst family in its respective domain. Current NMR research group at the National Institute of Clean-and-Low-Carbon Energy of China focuses on olefin polymerization catalysts using one of the world’s most advanced 700 MHz liquid NMR spectrometers equipped with a 10 mm helium-cooled cryoprobe. The relevant findings are expected to exert profound impacts on both industrial applications and fundamental academic research.

## Figures and Tables

**Table 1:** ^1^H chemical shifts in complexes (Ligands: CDCl_3_/CCl_4_; Complex **1** precatalyst: CDCl_3_, 60 °C; Complex **2** precatalyst: methanol-d_4_, 60 °C; 400 MHz).

Complex	Molecular Formula	Structure Label	^1^H Chemical Shift/ppm
Complex **1** (ligand)	C_21_H_17_F_2_N_3_	A–E	8.34 (A), 7.86 (B), 7.04 (C), 6.74 (D), 2.39 (E)
Complex **1** (precatalyst)	C_21_H_17_Cl_2_F_2_N_3_Ni	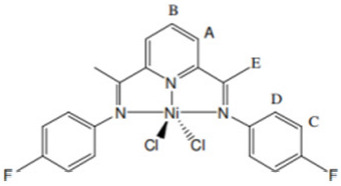	72.49 (A), 19.63 (B), 14.95 (C), 0.004 (D), −2.51 (E)
Complex **2** (ligand)	C_21_H_17_Br_2_N_3_	A–E	8.33 (A), 7.87 (B), 7.47 (C), 6.70 (D), 2.39 (E)
Complex **2** (precatalyst)	C_21_H_17_Cl_2_Br_2_N_3_Ni	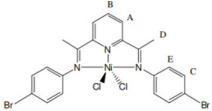	68.72 (A), 15.57 (B), 12.57 (C), −2.85 (D), −7.18 (E)

## Data Availability

No new data were created or analyzed in this study.
